# Comparative Evaluation of Dexmedetomidine and Magnesium Sulfate for Prevention of Postoperative Atrial Fibrillation in Patients of Coronary Artery Bypass Surgeries

**DOI:** 10.7759/cureus.41075

**Published:** 2023-06-28

**Authors:** Jui A Jadhav, Shrilekh Mankhair, Vivek Chakole

**Affiliations:** 1 Anaesthesiology, Jawaharlal Nehru Medical College, Datta Meghe Institute of Higher Education and Research, Wardha, IND

**Keywords:** coronary artery bypass grafting (cabg), off-pump coronary artery bypass graft surgery, atrial fib, atrial arrhythmia, intravenous magnesium sulfate, dexmedetomidine

## Abstract

Introduction

The main goal of this study is to compare the effects of dexmedetomidine and magnesium sulfate on preventing atrial fibrillation (AF) after off-pump coronary artery bypass graft (CABG) surgeries. AF is a type of irregular heartbeat that often occurs after heart surgery and can cause serious complications. We want to see which drug is more effective in reducing the risk of AF in patients who have had CABG surgeries without using a heart-lung machine.

Materials and methods

This was a randomized clinical study, conducted on patients of ASA classes III and IV who were the candidates for off-pump coronary artery bypass graft surgeries, which were conducted in the cardiac operating room from the period October 2020 to April 2021, at the Department of Anaesthesia, Jawaharlal Nehru Medical College, Sawangi, Meghe. All patients were aged between 30 and 85 years and with a left ventricular ejection fraction of above 40%.

Results

When the two groups were compared, the incidence of arrhythmias was more in group D (a group that received injection dexmedetomidine infusion), predominantly AF with an incidence of 50% more as compared to group M (a group that received injection magnesium sulfate infusion). When hemodynamic parameters were compared, events of bradycardia and hypotension were significantly higher in group D as compared to group M. The heart rate (HR), when compared between the two groups intraoperatively from the duration before induction of anesthesia till during sternum closure, has a significant p-value (0.0001). Similarly, when the mean arterial pressure (MAP) was compared between the two groups intraoperatively, significant hypotension was noted in group D (p-value = 0.0001). Postoperatively, in the intensive care unit when the HR and MAP were compared for 48 hours, bradycardia and hypotension were noted, but drastic changes in the mean values of the HR and MAP were not seen in both groups D and M.

Conclusion

When comparing the antiarrhythmic properties of the study drugs, it was observed that intraoperative and postoperative arrhythmias were less with magnesium sulfate as compared with dexmedetomidine. It was also found that there were higher events of hypotension and bradycardia in the dexmedetomidine group as compared to the magnesium sulfate group.

## Introduction

Arrhythmia is a common complication after heart surgery and is a major source of morbidity and mortality [1]. The most common type of irregular heartbeat is atrial fibrillation (AF) [2]. Even with better surgery methods, anesthesia, and heart protection during the operation, the number of patients who have AF after coronary artery bypass graft (CABG) surgeries has not gone down [3]. This study involves comparing the evaluation of dexmedetomidine and magnesium sulfate on the prevention of postoperative AF in patients undergoing off-pump CABG surgeries.

Dexmedetomidine is a short-acting drug that is given through a vein and works by activating alpha-2 receptors. Its role in preventing irregular heartbeats during surgery is not well-known. It inhibits the sympathetic nervous system excitation and reduces the release of norepinephrine, leading to a decrease in the concentration of catecholamine in the blood and maintaining the stability of circulation. It may help prevent tachycardia, high blood pressure, confusion, anxiety, and pain during and after surgery [4,5]. There have been many studies conducted, to determine whether dexmedetomidine prevents the occurrence of arrhythmias, but still it is not clear. Dexmedetomidine lowers the stress response by reducing serum catecholamine levels, thereby preventing tachycardia and hypertension during and after surgery. This study mainly aims to evaluate its property in preventing arrhythmias and to compare it with magnesium sulfate for the same [6]. There are side effects associated with dexmedetomidine, such as a reduction in heart rate (HR), systemic vascular resistance, cardiac output, and myocardial contractility. These side effects can be managed with intravenous injection of atropine, ephedrine, and volume infusion [7,8].

Magnesium sulfate is another drug commonly used in the postoperative period to prevent cardiac arrhythmias. Magnesium works by blocking calcium, regulating energy transfer, and stabilizing membranes. It has many effects on the body, does not affect the heart’s pumping ability much, and has a wide safety margin. These make intravenous magnesium a good choice for preventing AF after surgery [9]. Zwillinger was the first to describe the anti-arrhythmic effects of magnesium sulfate in 1935. Magnesium sulfate can help with different kinds of irregular heartbeats. Lately, many studies are looking at how magnesium sulfate affects AF [10]. Low magnesium levels in the blood can increase the risk of AF, even without other heart problems. Intravenous magnesium can treat this condition [11]. There have been no studies done till now comparing magnesium sulfate and dexmedetomidine in preventing cardiac arrhythmias, though, individually, these drugs were studied for the same. This study primarily aims to compare the efficacy of both drugs in preventing AF intraoperatively and postoperatively in patients who have undergone CABG surgeries. The secondary objective is to compare hemodynamic parameters, such as HR and mean arterial pressure (MAP), and to evaluate the side effects associated with the two drugs. 

## Materials and methods

Study setting and design

This was a randomized clinical study, conducted on patients who were candidates for off-pump CABG surgeries from October 2020 to April 2021, in the Department of Anaesthesia, Jawaharlal Nehru Medical College, Acharya Vinoba Bhave Rural Hospital (AVBRH), Sawangi, Meghe. Informed and written consent was taken from the patients participating in this study. The inclusion and exclusion criteria of the study were as follows:

Inclusion criteria:

1. Patients undergoing off-pump CABG surgeries.

2. ASA classes III and IV.

3. Age range of 30-85 years.

4. Ventricular EF more than 40%.

Exclusion criteria:

1. Patients with a previous history of AF.

2. History of previous cardiac surgeries, valvular heart diseases, and cardiomegaly.

3. Patients with renal failure (serum creatinine > 2.5 mg/dl).

4. Patients with hepatic dysfunction.

5. Patients with severe lung diseases.

6. Patients with pulmonary hypertension.

7. Recent history of myocardial infarction less than 15 days.

A total of 20 patients satisfying the inclusion criteria were included in the study. The patients were posted for off-pump CABG surgeries under general anesthesia. They were divided into two groups as follows: 

Group D: Patients received dexmedetomidine 0.5 ug/kg/hr infusion, started once the patient was induced and continued for two days in the cardiac intensive care unit (ICU). 

Group M: Patients received magnesium sulfate 20 ug/kg/min infusion, started once the patient was induced and continued for two days in the cardiac ICU. 

Preoperative Assessment

The day before the surgery, all patients were examined, and a thorough airway assessment was done. Patients with a history of hypertension received anti-hypertensive medication till the day of surgery, except angiotensin-converting enzyme inhibitors and angiotensin receptor blockers. All the patients were kept nil by mouth on the day of surgery as per standard ASA guidelines. 

Intraoperative Events

Patients were shifted to the operating room, and standard ASA monitors were connected. Baseline vital parameters were recorded. An 18 G intravenous cannula was secured. With adequate local anesthetic infiltration with lignocaine, a radial arterial line was secured for invasive arterial blood pressure monitoring. A right internal jugular central venous catheter was secured. All the patients were given general anesthesia. They were pre-medicated with Inj. midazolam 1 mg i/v and Inj. fentanyl 3 ug/kg body weight. Patients were induced with Inj. thiopentone 5 mg/kg body weight and paralyzed with Inj. vecuronium 0.1 mg/kg, following which they were intubated with an adequate-size endotracheal tube. Post this, an infusion of dexmedetomidine in group D patients and magnesium sulfate in group M patients was started and was continued for two days postoperatively in the ICU. Anesthesia was maintained with isoflurane and Inj. vecuronium. All the patients were monitored continuously with ECG, pulse oximetry, arterial blood pressure, and central venous pressure. When required, arterial blood gas sampling and activated clotting time monitoring were done.

Primary Endpoint

The primary endpoint of the study was considered the prevention of AF intraoperatively and postoperatively. The occurrence of AF was recorded. The occurrence of any other cardiac arrhythmias, postoperative hemodynamic instability, incidence of hyper magnesia, and side effects such as bradycardia associated with the two drugs was noted.

The primary objective of the study was to evaluate the efficacy of magnesium sulfate and dexmedetomidine in preventing arrhythmias, predominantly AF, intraoperatively and postoperatively for a period of two days.

The secondary objective was to compare hemodynamic parameters, such as HR and MAP, and to evaluate the side effects associated with the two drugs.

Statistical analysis

The data were analyzed utilizing the Chi-square test and Student T-test to compare the variables. The software was the Statistical Package for Social Sciences (SPSS) 17.0.

Ethical consideration

Written informed consent was obtained from each participant after a careful explanation of the concept and purpose of the study. The participants were ensured privacy and confidentiality. The study protocol was reviewed and approved by the DMIMS(DU)/IEC/2020-21/22.

## Results

Table 1 shows the frequency and percentage of different types of arrhythmias in the two groups of patients: groups M and D. The primary objective of the study of evaluating the occurrence of AF showed a 50% higher incidence of AF in group D as compared to group M. The p-value indicates the statistical significance of the difference between the groups. According to the table, the only arrhythmias that showed a significant difference between the groups were AF and bradycardia, both of which were more common in group D than in group M. The other types of arrhythmias did not show a significant difference between the groups.

**Table 1:** Distribution of patients in the two groups according to the occurrence of arrhythmias

Arrhythmias	Group M	Group D	p-value
Atrial Fibrillation	0 (0%)	5 (50%)	0.032
Atrial Flutter	0 (0%)	0 (0%)	0.17
Ventricular Fibrillation	1 (10%)	2 (20%)	0.33
Ventricular Flutter	0 (0%)	0 (0%)	0.17
Ventricular Tachycardia	0 (0%)	0 (0%)	0.17
Bradycardia	0 (0%)	5 (50%)	0.032
Asystole	0 (0%)	1 (10%)	0.32

Table 2 shows the mean and standard deviation of the HR in the two groups of patients: groups M and D. The variable was measured at different time points during a cardiac surgery procedure: before induction, during sternotomy, 30 min after induction, during distal anastomosis, during proximal anastomosis, and during sternum closure. The t-value and p-value indicate the results of a statistical test to compare the variable between the groups at each time point. According to the table, the HR was significantly lower in group D than in group M at all time points, except 30 minutes after induction and during distal anastomosis where there was no significant difference between the groups.

**Table 2:** Comparison of heart rates in the two groups

Heart Rate	Group M		Group D		t-value	p-value
	Mean	SD	Mean	SD		
Before Induction	90.21	9.56	88.21	8.21	5.26	0.0001
During Sternotomy	86.21	5.92	84.21	4.21	4.21	0.0001
30 min After induction	82.21	4.53	80.56	1.32	4.29	0.046
During Distal Anastomosis	85.56	3.51	83.19	2.31	4.13	0.031
During Proximal Anastomosis	76.59	5.31	74.56	5.39	5.21	0.0001
During Sternum Closure	82.21	1.56	80.19	0.59	5.31	0.0001

According to Table 3, the mean arterial pressure was significantly higher in group M than in group D at all time points. The mean arterial pressure showed a significant difference at all steps, except 30 minutes after induction and during distal anastomosis.

**Table 3:** Comparison of the mean arterial pressure in the two groups

Mean Arterial Pressure	Group M		Group D		t-value	p-value
	Mean	SD	Mean	SD		
Before Induction	115.7	10.50	111.2	11.21	5.21	0.0001
During Sternotomy	74.5	11.21	72.36	9.51	6.21	0.0001
30 min After induction	86.2	5.92	82.59	4.92	4.21	0.041
During Distal Anastomosis	64.5	6.21	62.4	6.96	4.31	0.039
During Proximal Anastomosis	58.6	5.61	54.56	9.21	6.19	0.0001
During Sternum Closure	78.6	5.9	72.56	5.21	5.39	0.0001

Table 4 shows the mean values of the HR and MAP in group D patients at different time points during ICU monitoring after a cardiac surgery procedure. The time points are one hour, two hours, three hours, four hours, five hours, six hours, 12 hours, 18 hours, 36 hours, and 48 hours. According to the table, the HR showed a slight increase from one hour to five hours, then a slight decrease from six hours to 12 hours, and then a slight increase again from 18 hours to 48 hours. The mean arterial pressure showed a slight decrease from one hour to two hours, then a slight increase from three hours to 36 hours, and then a slight decrease from 36 hours to 48 hours. The HR and MAP did not show any drastic changes over time in group D patients.

**Table 4:** Distribution of intensive care unit monitoring for group D

Group D	Heart Rate (HR/min)	Mean Arterial Pressure (MAP/mmHg)
1 hour	69.19	71.22
2 hours	75.2	70.26
3 hours	71.21	72.12
4 hours	74.32	72.21
5 hours	75.52	72.41
6 hours	71.36	73.32
12 hours	64.23	72.16
18 hours	65.15	74.26
36 hours	66.31	75.06
48 hours	69.16	74.24

Table 5 shows the mean values of the HR and MAP in group M patients at different time points during ICU monitoring after a cardiac surgery procedure. The time points are one hour, two hours, three hours, four hours, five hours, six hours, 12 hours, 18 hours, 36 hours, and 48 hours. According to the table, the HR showed a slight decrease from one hour to five hours and then a slight increase from six hours to 48 hours. The mean arterial pressure showed a slight decrease from one hour to two hours, then a slight increase from three hours to six hours, then a slight decrease from 12 hours to 18 hours, and then a slight increase from 36 hours to 48 hours. The HR and MAP did not show any drastic changes over time in group M patients.

**Table 5:** Distribution of intensive care unit monitoring for group M

Group M	Heart Rate (HR/min)	Mean Arterial Pressure (MAP/mmHg)
1 hour	89.29	77.12
2 hours	85.26	75.56
3 hours	81.21	76.12
4 hours	84.36	75.21
5 hours	78.52	76.41
6 hours	81.36	77.32
12 hours	84.43	76.26
18 hours	85.35	73.21
36 hours	86.31	75.36
48 hours	89.56	76.54

## Discussion

Arrhythmia is a common complication after heart surgery and is a major source of morbidity and mortality [12]. AF is the most common arrhythmia encountered by anesthesiologists during intraoperative and postoperative periods. Arrhythmias are more frequent in patients with coronary artery disease [13]. The commonest cause of arrhythmias in the pre-bypass period is the surgical manipulation of the heart such, as during placement of sutures, cannulation, and vent placement [14]. Other causes include preexisting cardiac arrhythmias, increased catecholamine levels due to light anesthesia, electrolyte abnormalities such as hypokalemia and hypomagnesemia, hypotension, hypoxemia, hypertension, and myocardial ischemia [15].

Both on- and off-pump CABG procedures, which are performed while the patient's heart is still beating, might result in arrhythmias. The most frequent reason for arrhythmias during on-pump CABG procedures is when the heart is being operated on during the pre-bypass phase. Patients who are hypomagnesemic and experience arrhythmias benefit from magnesium treatment. Arrhythmias can have both small and significant hemodynamic effects [16].

Supraventricular tachycardia, which includes AF and atrial flutter, is an arrhythmia that might have mild hemodynamic effects or disruptions. Treatment options include vagal maneuvers, beta-blockers, calcium blockers, adenosine, and digoxin in addition to stopping mechanical irritation. Evaluation and treatment are also required for premature ventricular contractions. Lidocaine, amiodarone, and beta blockers are to be used to relieve mechanical irritation and stop it altogether [17].

For the arrhythmias that are seriously impairing hemodynamics such as ventricular tachycardia, ventricular fibrillation, and atrial arrhythmias, cardioversion and defibrillation must be started.

1. When the chest is open, small paddles are placed directly on the surface of the heart to induce internal cardioversion. For cardioversion, low energy levels of 10-25 J are required.

2. Defibrillation requires the same level of energy in a non-synchronized mode.

3. With the chest closed, a regular paddle size is used to induce external cardioversion. Twenty-five to 300 J of energy is required.

4. Defibrillation should be started with 300 J.

Arrhythmias during off-pump CABG are those that happen in CABG operations on beating hearts. Anaesthesiologists have a difficult time controlling intraoperative hemodynamic changes during beating heart procedures [18]. They have to manage intraoperative myocardial ischemia when coronary blood flow is cut off during grafting in addition to maintaining hemodynamic stability during heart enucleation, which is required to access each coronary artery.

In order to ensure bloodless anastomotic circumstances, coronary artery cross-clamping is done during off-pump coronary artery bypass (OPCAB). This causes a brief period of myocardial ischemia that manifests as ST-segment elevation and new regional wall motion abnormalities [19]. When the non-occlusive RCA (right coronary artery) is clamped, there is severe ischemia that can lead to serious arrhythmias such as full AV block (atrioventricular block) because the blood supply to the AV node is cut off.

By generating arterial vasodilation, reducing hypertension, and preventing coronary vasospasm, magnesium plays a critical part in OPCAB in preventing arrhythmias. Magnesium sulfate must be used to pump CABG procedures as an antiarrhythmic medication. Patients undergoing cardiopulmonary bypass (CPB) graft surgeries frequently suffer from magnesium depletion. It is advised to use universal Cardioplegia solutions that contain magnesium, and adding Mg to the pump is also crucial [20].

Dexmedetomidine and magnesium sulfate's effectiveness in preventing arrhythmias, particularly AF, both during surgery and afterward for patients undergoing coronary artery bypass graft procedures, was examined in this study. Dexmedetomidine is a highly selective alpha 2 receptor agonist that provides calming, antianxiety, and pain-relieving benefits without causing respiratory depression. It is being used more frequently to sedate patients undergoing heart surgery. Dexmedetomidine is a great medication for avoiding arrhythmias since it possesses sympatholytic, anxiolytic, analgesic, and sedative characteristics. However, bradycardia and hypotension are discovered to be two of its adverse effects. After heart surgery, it lessens myocardial ischemia-reperfusion injury and improves cardiac muscle perfusion. The inflammatory reaction brought on by bypass operations may also alter the electrophysiological and structural makeup of the atrium layers and enhance the sensitivity of AF. Dexmedetomidine lowers catecholamine levels and guards against arrhythmias It causes alterations in the calcium flow across the membrane of cardiac muscle cells and increases vagus nerve activity, both of which prolong the time that repolarization lasts and result in an effective refractory period [21].

Magnesium sulfate was another medication examined in this research. Without causing any adverse effects such as bradycardia and hypotension, it was discovered in the current study that it dramatically decreased the occurrence of arrhythmias in patients who underwent coronary artery bypass graft procedures. Thus, it was discovered that it shortens hospital stays.

It has been discovered that magnesium sulfate has calcium-antagonistic characteristics. It also functions as a membrane stabilizer and an energy transfer regulator. It has fewer inotropic side effects and a wide therapeutic window when administered intravenously. AF can be prevented with a safe and effective medication thanks to magnesium sulfate. Magnesium sulfate exhibits alpha-adrenergic antagonist properties in addition to calcium antagonist properties. Magnesium has no effect on the inotropic effects of epinephrine. It possesses sympatholytic qualities and produces arterial vasodilation in addition to preventing coronary vasospasm, myocardial ischemia, and arrhythmias, and improving cardiac outcomes [22]. Critically ill patients, diabetics, alcoholics, and anyone taking extended diuretics all frequently lack enough magnesium. Since hypomagnesemia puts cardiac patients at risk for arrhythmias, it should be addressed before surgery [23]. In our study, a magnesium infusion was started as soon as the patients were sedated to prevent arrhythmias during and after surgery.

Monitoring serum magnesium levels is crucial. Magnesium poisoning can cause cardiac arrest and is dangerous. Cardiovascular decompensation should not play a significant impact until the plasma concentration is higher than 12 mmol/L. Magnesium sulfate and dexmedetomidine have been used in several studies to treat AF in patients scheduled for CABG surgery. Based on this study conducted, magnesium sulfate was found to have significantly reduced the incidence of arrhythmias, predominantly AF, in the patients who underwent elective CABG surgeries. Magnesium sulfate when compared with dexmedetomidine, decreased the incidence of arrhythmias by up to 50%. In comparison to this study, Naghipour et al. conducted a randomized placebo-controlled study of the effect of magnesium sulfate on the reduction of post-cardiac surgery arrhythmias in 160 patients. Eighty patients received 30 mg/kg of magnesium sulfate in 500 milliliters of normal saline, and the remaining 80 patients received 500 milliliters of normal saline. It was concluded that there was a significant decrease in the incidence of post-cardiac surgery arrhythmia and thereby reducing hospital stay [9].

Another study was conducted by Soltani et al. on the effects of dexmedetomidine in preventing heart arrhythmias in off-pump surgeries on 76 patients. Patients were randomly assigned into two groups of intervention. The first group received an infusion of 0.5 microgram/kilogram/hour for induction, followed by the same rate of infusion until the end of the surgery. The control group received a saline infusion. It was concluded that dexmedetomidine was found to reduce the incidence of arrhythmias in off-pump CABG surgeries significantly [6].

In the present study, when MAP and HR were observed as variables, it was found that there was a higher prevalence of hypotension and bradycardia in the dexmedetomidine group as compared to the magnesium sulfate group. Soltani et al., observing hemodynamics parameters of HR and MAP, found that the control group which received normal saline showed higher values of MAP and HR during surgery and ICU admission compared to the intervention group, which received dexmedetomidine [6].

Another study was conducted on 20 patients by Kabukçu et al. on the effects of intraoperative dexmedetomidine administration on hemodynamics in coronary artery bypass surgery. It was found that with dexmedetomidine infusion, HR and MAP were found to be moderately decreased compared to baseline values, and no events of bradycardia and hypotension were noted. Thus, it was concluded that dexmedetomidine can be safely used in CABG operations providing stable hemodynamic status [24].

The main advantage of our study was to compare the antiarrhythmic properties of magnesium sulfate and dexmedetomidine, rather than individually assessing these two drugs. Another advantage of this study was that dexmedetomidine was studied for its antiarrhythmic properties rather than its known properties of sedation and analgesia.

Limitations of the study include the sample size being reduced because of the COVID-19 pandemic and the exclusion of patients with previous history of AF, previous cardiac surgeries and valvular heart diseases, hepatic dysfunction, renal failure, severe lung diseases, pulmonary hypertension, recent history of myocardial infarction of less than 15 days, and those undergoing on-pump CABG surgeries.

## Conclusions

When comparing the antiarrhythmic properties of the two drugs, it was observed that intraoperative and postoperative arrhythmias were less with magnesium sulfate as compared with dexmedetomidine. It was also found that there was a higher incidence of hypotension and bradycardia associated with dexmedetomidine as compared to magnesium sulfate.
